# Impact of nausea/vomiting on EQ-5D-5L utility scores in patients taking iron preparations for heavy menstrual bleeding or anemia

**DOI:** 10.1186/s12905-023-02652-1

**Published:** 2023-09-21

**Authors:** Kyoko Ito, Yuko Mitobe, Ryo Inoue, Mikio Momoeda

**Affiliations:** 1 Medical Affairs Department, Torii Pharmaceutical Co., Ltd., 3-4-1 Nihonbashi-Honcho, Chuo-ku, Tokyo, 103-8439 Japan; 2 Aiiku Maternal and Child Health Center, Aiiku Hospital, 1-16-10 Shibaura, Minato-ku, Tokyo, 105-8321 Japan

**Keywords:** Iron preparations, Nausea, Vomiting, EQ-5D-5L utility scores, Heavy menstrual bleeding, Anemia

## Abstract

**Background:**

The purpose of this study was to establish an estimating equation to predict the 5-level EQ-5D version (EQ-5D-5L) utility score in patients taking iron preparations for heavy menstrual bleeding (HMB) or anemia and to evaluate whether the presence of nausea or vomiting was a significant predictor of EQ-5D-5L-based quality of life.

**Methods:**

A cross-sectional survey was conducted to collect EQ-5D-5L utility scores and other patient reported outcomes from 385 patients taking iron preparations for HMB or anemia who were selected from the disease patient panel. Using the utility scores as objective variables, explanatory variable candidates were selected considering correlations, multicollinearity, and clinical validity. Predicting models were constructed using regression-based models (linear model, generalized linear model (GLM), Tobit model). Stepwise regression method was applied for selecting statistically significant (p < 0.05) predictors. Goodness-of-fit of models were assessed by mean absolute error and mean squared error (MSE).

**Results:**

The EQ-5D-5L utility scores (mean ± standard deviation) of 96 patients with nausea/vomiting and 289 patients without nausea/vomiting were 0.67 ± 0.16 and 0.84 ± 0.14, respectively (p < 0.001). The presence of nausea/vomiting was shown to be the most significant factor reducing the utility score in the statistical models using the explanatory variable candidates selected in the study. As the results of the goodness-of-fit test, GLM with the smallest MSE was selected to establish the estimating equation.

**Conclusion:**

The estimating equation to predict the EQ-5D-5L utility scores in patients taking iron preparations for HMB or anemia was established. The presence of nausea/vomiting was found to be a factor significantly reducing utility scores, with a decrement of the value estimated to be -0.117.

**Trial registration:**

UMIN000045700 (http://www.umin.ac.jp/ctr/). Registered on October 11, 2021.

**Supplementary Information:**

The online version contains supplementary material available at 10.1186/s12905-023-02652-1.

## Introduction

The World Health Organization (WHO) reported that global anemia prevalence in women aged 15 to 49 years was 29.9% in 2019 and failure to reduce anemia prevalence may impair health and quality of life (QOL) of those women [1]. The common cause of anemia in females is iron deficiency. It is associated with insufficient dietary iron intake or absorption, an increase in iron demand during pregnancy, and a menstruation-related increase in iron loss [2]. Of these factors, heavy menstrual bleeding (HMB) is noted in approximately 18 to 38% of females of reproductive age [3]. HMB, iron deficiency and iron deficiency anemia (IDA) have related conditions, and that negatively impact QOL [4]. Reproductive-aged women worldwide are frequently suffered by these conditions and our society needs to recognize and address this issue.

In patients with HMB or anemia, treatment with iron preparations is performed in addition to the treatment of a primary disease for HMB and lifestyle modification. However, gastrointestinal symptoms, including nausea and vomiting, often appear as adverse events/reactions to iron preparations. It was reported that there was a significant correlation between these gastrointestinal symptoms, as adverse reactions, and a reduction in patients’ drug adherence [5]. In a meta-analysis of gastrointestinal issues with oral iron, ferrous sulfate, which is a widely used oral iron preparation overseas, significantly increased risk of gastrointestinal symptoms versus placebo with an odds ratio (OR) of 2.32 [95% confidence interval (CI) : 1.74–3.08] and versus intravenous iron with an OR of 3.05 (95% CI : 2.07–4.48) [6]. A study of ferrous sulfate, in pregnant women with IDA showed that gastrointestinal symptoms occurred as adverse events, and that the incidences of nausea and vomiting were 46.2% and 28.2%, respectively [7]. An intravenous (IV) iron preparation (ferric carboxymaltose) also induces gastrointestinal symptoms as adverse reactions, although the relative risk is lower than that of oral iron preparations [8]. Adverse events/reactions regarding nausea/vomiting related to these iron preparations may influence QOL of patients with HMB or anemia.

Cost-effectiveness evaluation (CEE) is applied for policies in countries around the world to prioritize various medical services for adequate allocation in a limited budget. Public organizations responsible for these assessments, including the National Institute for Health and Care Excellence (NICE) in England, exist in many countries. In Japan, a public evaluation organization, the Center for Outcomes Research and Economic Evaluation for Health (C2H), was also established, and CEE was officially institutionalized from 2019. In the analysis guideline for CEE in Japan [9], the quality-adjusted life year (QALY), which integrates life years and QOL, is recommended as an outcome. In this guideline, it is recommended that, as a rule, the utility score measured using the preference-based measure (PBM) should be used when calculating the QALY. A representative measure is 5-level EQ-5D version (EQ-5D-5L). It is recommended that EQ-5D-5L should be adopted as the first choice when newly collecting QOL values in Japan for CEE.

Therefore, to conduct a CEE in accordance with the analysis guideline for assessing the value of treatment for patients with nausea/vomiting due to taking iron preparations for HMB or anemia, it is essential to determine how much this nausea/vomiting impact on the patient’s QOL value, i.e., to generate the disutility value due to nausea/vomiting as measured by EQ-5D-5L.

Several studies evaluated the relationship between IDA or nausea/vomiting and QOL. Ando et al. followed up 92 females with IDA (6.0 < Hb < 11.0 g/dL, serum ferritin < 20 ng/mL) for 3 months after the start of oral iron preparation (sodium ferrous citrate, 100 mg/day) administration, and evaluated the QOL using the 36-item short-form health survey (SF-36). They reported that there were significant increases in all domains but role-emotional (physical functioning, role-physical, social functioning, mental health, bodily pain, vitality, and general health perception) [10]. Peuranpää et al. assessed the QOL for females receiving HMB treatment using the RAND 36-item health survey (RAND-36), EQ-5D, and visual analogue scale (VAS), and reported that the baseline EQ-5D utility scores in two groups with an Hb level of < 12 g/dL and ≥ 12 g/dL were 0.76 and 0.77, respectively, and that there were increases in the QOL score after 6 and 12 months in the two groups [11]. Bai et al. evaluated the association between nausea/vomiting/fatigue and the QOL assessed using the 12-item short-form health survey (SF-12) in 5,079 women in early pregnancy, and indicated that these symptoms reduced the physical- and mental-component scores of SF-12 [12]. These studies suggest that nausea/vomiting related to iron preparation administration to patients with HMB or anemia influence their QOL. However, to our knowledge, no study has evaluated it, and no studies investigated the impact of nausea/vomiting associated with taking iron preparations on QOL in patients with HMB or anemia.

In this study, we evaluated the QOL using the EQ-5D-5L utility score in patients taking iron preparations for HMB or anemia, and collected information on the presence of nausea/vomiting, other patient reported outcomes (PROs) and characteristics to establish an estimating equation for predicting the EQ-5D-5L utility score based on the hypothesis that the presence of nausea/vomiting related to the administration of iron preparations may reduce the QOL. Next, we assessed whether the presence of nausea/vomiting related to the administration of iron preparations is a significant predictive factor that influences EQ-5D-5L utility scores in the patients taking iron preparations for HMB or anemia from the estimating equation.

## Materials and methods

### Study design and participants

This study was a cross-sectional web-based survey questionnaires on a personal computer, a cell phone or a tablet, and the data were collected from patients taking iron preparations for HMB or anemia who were selected from the disease patient panel produced by INTAGE Healthcare Inc., (INTAGE, Tokyo, Japan). INTAGE annually investigates “current status of symptoms”, “therapeutic diseases”, and “drugs in use” with about 500,000 healthcare consumers/patients nationwide [13]. Based on the results of the pre-web-based survey conducted to confirm whether a questionnaire, which is a screening tool for patients who were taking iron preparations for HMB or anemia, was understood appropriately by patients and the feasibility of the study in patients with HMB or anemia who were registered in the disease patient panel (n = 2,818), including the prevalence of taking iron preparations and having nausea or vomiting. The target number of patients to be enrolled was set at 300, consisting of 50 patients with nausea/vomiting and 250 patients without nausea/vomiting. Patients taking iron preparations for HMB or anemia do not always take them every day. Therefore, in this study, the patients taking iron preparations were defined as those who had taken iron preparations at least within the past 3 months. After received the web-based survey invitation, patients who were registered as having gynecological related disorders in the disease patient panel, were screened using the following criteria. The eligible patients were defined by meeting the following four criteria: (i) have HMB or anemia, (ii) have taken iron preparations at least within the past 3 months, (iii) 20 years of age or older at the time of the survey and (iv) have access to an internet-capable device (computer, smartphone, or tablet) and are able to operate it, or have the cooperation of someone who can operate it. Patients who did not agree to participate in this study were excluded from this survey. The survey period was October 22–25, 2021, using a one-time web-based questionnaire and the anonymity of participants was required and guaranteed by INTAGE. The response data were recorded and collected electronically, and then INTAGE stored, accumulated, anonymized, and cleaned them. This web-based survey was designed that participants were not able to finish and submit their response data unless they completed the questionnaire in a fixed order. Therefore, there were no missing data.

The survey items included a questionnaire of EQ-5D items (relating to the 5 dimensions of mobility, self-care, usual activities, pain/discomfort, and anxiety/depression), sex, age, and the following items: primary disease (endometriosis, uterine myoma, adenomyosis uteri, endometrial polyps, dysmenorrhea, premenstrual syndrome (PMS), HMB, others), medication use (low-dose oral contraceptives, iron preparations (IV, oral, oral supplements), estrogen preparation, others), whether during menstruation period, presence of current symptoms (nausea, vomiting, nausea/vomiting, PMS, menstrual pain, menstrual symptoms, anemia). The timing of nausea or vomiting was the day patients answered the questionnaire, the frequency of nausea or vomiting was assessed by an open-ended question of “From how many days ago?”, the frequency of vomiting within 24 hours was assessed by an open-ended question of “How many times in a day?” and the severity of nausea was assessed by the question of “How terrible is your symptom (0 − 10)?”, respectively. These outcome measures may influence the QOL of the patients in this study from a clinical perspective and were determined after consultation with a physician specialized in obstetrics and gynecology.

The primary diseases were defined based on the options and the basis of self-report that the patients responded to as “diseases currently being treated by a gynecologist.” Medication use was defined as having medication if responding that a patient had taken drugs at least within the past 3 months. If at least one of the responses of “nausea” or “vomiting” was selected as a current symptom, it was defined “with nausea/vomiting”. The patients were defined as “currently having menstrual symptoms” if one of the responses of “during menstrual period” or “currently having symptoms of PMS” was selected. If at least one of the following responses was selected as a current symptom: “lightheadedness”, “shortness of breath”, “dizziness”, “lethargy”, or “tiredness”, the patient was defined as “currently having anemia”. HMB was defined as having HMB if the primary disease was HMB or at least one of the following responses was selected as the menstrual situation: “the amount of menstrual blood is so large that a regular sanitary napkin does not last for an hour,” “there are large blood clots like liver mixed with the menstrual blood,” or “the amount of menstrual blood is so large that it interferes with daily life such as frequently soiling bedding and clothes and making work impossible.”

The EQ-5D-5L is a generic preference-based health state utility scale which includes a health descriptive system based on 5 dimensions. The dimensions cover mobility, self-care, usual activities, pain/discomfort, and anxiety/depression, and are characterized by 5 levels (i.e., no problems, slight problems, moderate problems, severe problems, and unable to). The responses of the EQ-5D-5L were converted to Japanese EQ-5D-5L utility scores using the coefficient and intercept estimated by time-trade-off (TTO) data reported by Shiroiwa et al. [14].

This study was approved by the ethics review committee, “Non-Profit Organization MINS Institutional Review Board” on September 16, 2021, and clinical trial registration was made on October 11, 2021 (UMIN000045700). Before this study was conducted, electronic informed consent was obtained from all the subjects.

### Statistical analysis

The following procedure was used to confirm variables overlapped or correlated with the EQ-5D-5L utility scores.

#### Step 1. Confirmation of the relationships between the objective variable and each explanatory variable

EQ-5D-5L utility scores were used as objective variables, and a univariate regression analysis was performed on each of the explanatory variables to confirm the relationships between the objective variable and each explanatory variable. However, to avoid missing important variables due to confounding effects, variable selection based on the results of the univariate regression analysis was not performed, but only confirmation of the relationships was conducted.

#### Step 2. Evaluation of multicollinearity

The correlation coefficient (r) and Variance Inflation Factor (VIF) between all the explanatory variables were calculated. Among variables for which r was greater than 0.7 or VIF was greater than 5 and considered to be multicollinear between variables, one explanatory variable candidate was selected based on the results of the univariate regression analysis in Step 1, its clinical importance, and versatility in the estimation of the EQ-5D-5L utility scores.

#### Step 3. Assessment of clinical validity

The explanatory variable candidates were narrowed down in terms of the clinical validity.

Predicting models were constructed using three regression-based types of statistical algorithms. The linear model is applied widely in order to estimate unknown parameters in a linear regression model by minimizing the sum of squared errors from the data. The generalized linear model (GLM) method can be applicable for the non-normal distribution of dependent variables and predict the disutility value (1-(EQ-5D-5L utility score)). The GLM estimated using a Gaussian distribution with a link function of log form was shown to be appropriate for this study. Since the dependent variable showed a ceiling effect, the Tobit model was also used in this study for addressing the censored nature of EQ-5D-5L data. For selecting the final statistically significant (p < 0.05) predictors, the stepwise regression method was applied.

### Goodness-of-fit

As no external data were available for this study, the hold-out method was used for validating the alternative models. That is, all samples were randomly divided into two groups: 70% of the data (estimation dataset) were selected to create the predicting models. The remaining 30% (validation dataset) were set aside to verify the goodness-of-fit of the model. The goodness-of-fit of the fitting model was tested with two commonly used indicators: mean absolute error (MAE) and mean squared error (MSE). Moreover, the Spearman rank correlation coefficient was also examined to determine the strength of correlations between the predicted values and observed values.

The assessment process of the goodness-of-fit are explained briefly. (i) Data partitioning: the samples were divided into two groups at a ratio of 7:3, with each group as an estimation dataset and a validation dataset, (ii) Estimation and prediction: the parameters of the model were estimated using the estimation dataset, and the predicted values were calculated by inputting the validation dataset into the model, (iii) Calculation of indicators: the MAE and MSE were calculated to assess the goodness-of-fit of the models. The model with the smallest MSE was selected, and the equation of the EQ-5D-5L utility scores was reconstructed using the entire dataset.

All statistical analyses were conducted in SAS version 9.4 (SAS Institute Inc, Cary, NC, USA) or Stata version 15.0 (Stata Corp LP, College Station, TX, USA). P-values represent the p-values resulting from the Pearson’s chi-square test for discrete variables and the t-test for continuous variables, and p-values of < 0.05 were considered significant.

## Results

### Patient characteristics

A total of 385 patients were enrolled in the web-based survey. Of the 385 patients, 96 were patients had nausea/vomiting, of which 94 had nausea and 27 had vomiting. All the patients were female with a mean age of 41.6 ± 7.7 years (mean ± standard deviation (SD)). Descriptive statistics for the questionnaire responses are summarized in Additional file 1.

### EQ-5D-5L utility score

**Fig. 1:**
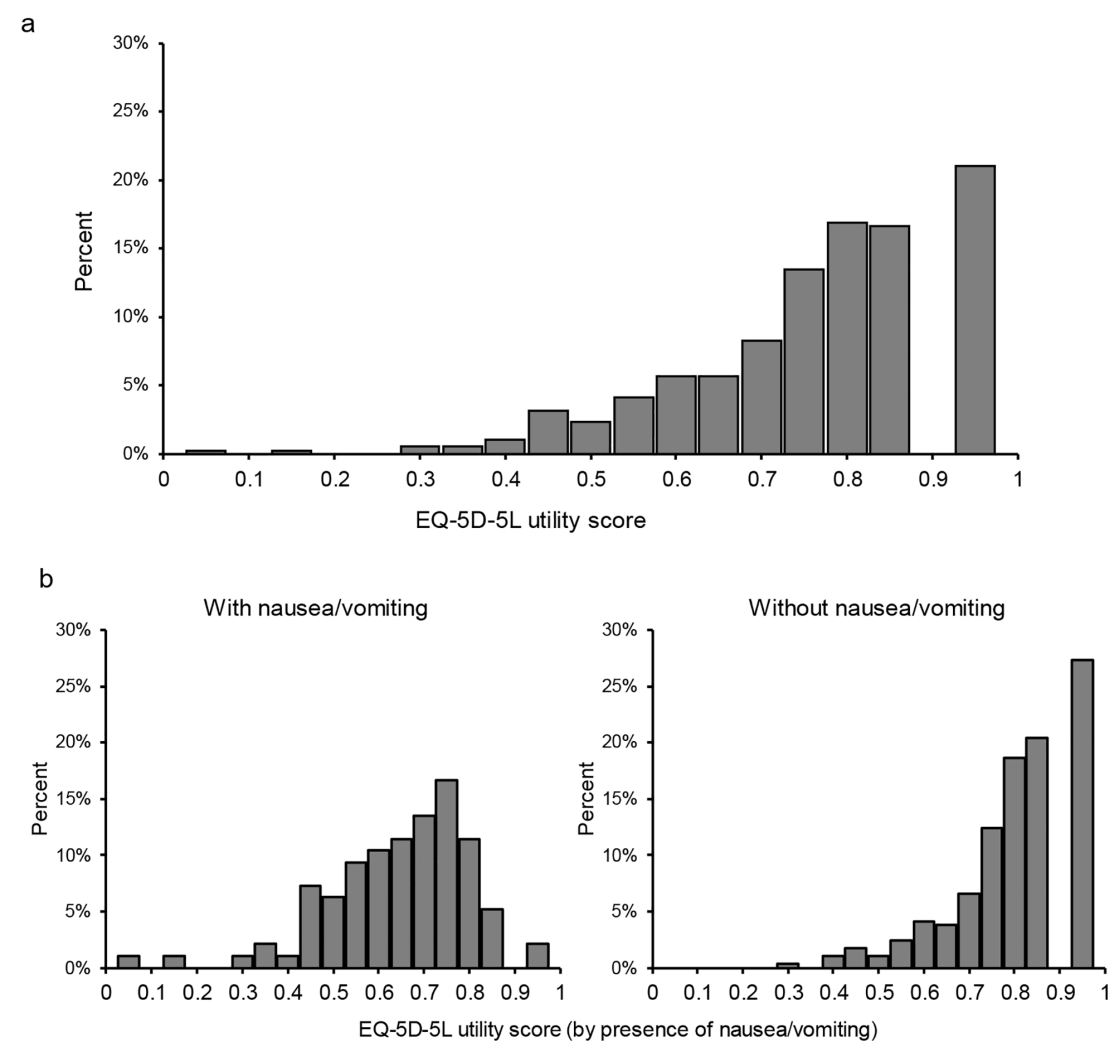
Distribution of EQ-5D-5L utility scores. **a** All (N = 385) = 0.79 ± 0.16 **b** Presence of nausea/vomiting (Yes = 96, No = 289), Presence of nausea/vomiting = 0.67 ± 0.16 and Absence of nausea/vomiting = 0.84 ± 0.14, p < 0.001, t-test between the groups with or without nausea/vomiting, Data are presented as mean ± SD

The EQ-5D-5L utility score (mean ± SD) of 385 patients was 0.79 ± 0.16. The EQ-5D-5L utility scores (Mean ± SD) of 96 patients with nausea/vomiting and 289 patients without nausea/vomiting were 0.67 ± 0.16 and 0.84 ± 0.14, respectively (Table 1). Figure 1a shows the distribution of the EQ-5D-5L utility scores in all patients. The EQ-5D-5L utility scores of patients with nausea/vomiting were significantly lower than those without nausea/vomiting (p < 0.001) (Fig. 1b). Descriptive statics for all patients and patients by the presence of nausea/vomiting of the EQ-5D-5L utility score and EQ-5D-5L domains are summarized in Table 1. In all five domains, there were significant differences (p < 0.001) in the distribution of responses (5-levels) by presence of nausea/vomiting. The results showed a higher percentage of lower level (i.e., worse level) responses in the patients with nausea/vomiting.

**Table 1:** Descriptive statistics for the EQ-5D-5L utility score and EQ-5D-5L domains

Variable description	All	Presence of nausea/vomiting		
	Total N = 385	No N = 289	Yes N = 96	p-value
EQ-5D-5L utility score	0.79 ± 0.16	0.84 ± 0.14	0.67 ± 0.16	< 0.001
VAS score	59.9 (24.4)	65.3 (22.8)	43.5 (21.7)	< 0.001
Mobility				
No problems	256 (66.5%)	221 (76.5%)	35 (36.5%)	< 0.001
Slight problems	91 (23.6%)	52 (18.0%)	39 (40.6%)	
Moderate problems	32 (8.3%)	13 (4.5%)	19 (19.8%)	
Severe problems	6 (1.6%)	3 (1.0%)	3 (3.1%)	
Unable to	0 (0.0%)	0 (0.0%)	0 (0.0%)	
Self-care				
No problems	333 (86.5%)	262 (90.7%)	71 (74.0%)	< 0.001
Slight problems	38 (9.9%)	23 (8.0%)	15 (15.6%)	
Moderate problems	10 (2.6%)	2 (0.7%)	8 (8.3%)	
Severe problems	4 (1.0%)	2 (0.7%)	2 (2.1%)	
Unable to	0 (0.0%)	0 (0.0%)	0 (0.0%)	
Usual activities				
No problems	222 (57.7%)	196 (67.8%)	26 (27.1%)	< 0.001
Slight problems	126 (32.7%)	75 (26.0%)	51 (53.1%)	
Moderate problems	29 (7.5%)	14 (4.8%)	15 (15.6%)	
Severe problems	7 (1.8%)	4 (1.4%)	3 (3.1%)	
Unable to	1 (0.3%)	0 (0.0%)	1 (1.0%)	
Pain/discomfort				
No pain or discomfort	151 (39.2%)	138 (47.8%)	13 (13.5%)	< 0.001
Slight pain or discomfort	157 (40.8%)	119 (41.2%)	38 (39.6%)	
Moderate pain or discomfort	62 (16.1%)	27 (9.3%)	35 (36.5%)	
Severe pain or discomfort	12 (3.1%)	5 (1.7%)	7 (7.3%)	
Extreme pain or discomfort	3 (0.8%)	0 (0.0%)	3 (3.1%)	
Anxiety/depression				
Not anxious or depressed	140 (36.4%)	128 (44.3%)	12 (12.5%)	< 0.001
Slightly anxious or depressed	152 (39.5%)	112 (38.8%)	40 (41.7%)	
Moderately anxious or depressed	53 (13.8%)	30 (10.4%)	23 (24.0%)	
Severely anxious or depressed	28 (7.3%)	15 (5.2%)	13 (13.5%)	
Extremely anxious or depressed	12 (3.1%)	4 (1.4%)	8 (8.3%)	

The numbers in the table represent N (%) for discrete variables and mean (SD) for continuous variables. P-values represent the p-value resulting from the Pearson’s chi-square test for discrete variables and the t-test for continuous variables

EQ-5D-5L, 5-level EQ-5D version; SD, standard deviation; VAS, visual analogue scale, 0-100 scale to indicate overall health

**Table 2:** Results of Multivariate analysis a) Linear Model (LM)

EQ-5D-5L utility score	Coefficient	SE	t	P > t	95% Confidence Interval	
Symptom_nausea/vomiting	-0.12198	0.01639	-7.44	0	-0.15421	-0.08975
Symptom_anemia	-0.09218	0.018764	-4.91	0	-0.12908	-0.05529
Symptom_menstrual pain	-0.08576	0.017225	-4.98	0	-0.11962	-0.05189
Symptom_PMS	-0.05925	0.017211	-3.44	0.001	-0.09309	-0.02541
Primary disease_PMS	-0.04641	0.019581	-2.37	0.018	-0.08491	-0.00791
Constant term	0.945575	0.016834	56.17	0	0.912476	0.978675

When the explanatory variable increases by 1, the EQ-5D-5L utility score can be interpreted as increasing by that coefficient

For example, if all the other conditions are the same, but the presence or absence of nausea/vomiting is different, it can be interpreted as 0.12198 lower for those with nausea/vomiting compared to those without nausea/vomiting

EQ-5D-5L, 5-level EQ-5D version; PMS, premenstrual syndrome; SE, standard error

**Table:** b) Generalized Linear Model (GLM)

EQ-5D-5L utility score	Coefficient	SE	z	P > z	95% Confidence Interval	
Symptom_nausea/vomiting	-0.165	0.023076	-7.15	0	-0.21022	-0.11977
Symptom_anemia	-0.10595	0.021203	-5	0	-0.1475	-0.06439
Symptom_menstrual pain	-0.10562	0.022635	-4.67	0	-0.14998	-0.06125
Symptom_PMS	-0.07073	0.021922	-3.23	0.001	-0.1137	-0.02777
Primary disease_PMS	-0.06672	0.027276	-2.45	0.014	-0.12018	-0.01326
Constant term	-0.05267	0.018312	-2.88	0.004	-0.08856	-0.01678

When the explanatory variable increases by 1, the EQ-5D-5L utility score can be interpreted as increasing by that coefficient x 100%

For example, if all the other conditions are the same, but the presence or absence of nausea/vomiting is different, it can be interpreted as 16.5% lower for those with nausea/vomiting compared to those without nausea/vomiting

EQ-5D-5L, 5-level EQ-5D version; PMS, premenstrual syndrome; SE, standard error

**Table:** The difference in the EQ-5D-5L utility score is shown below as the mean of the patient’s reduced EQ-5D-5L utility score

Variable description	Coefficient	Variable mean	X = 1	X = 0	Difference
Symptom_nausea/vomiting	-0.165	0.249	0.654231	0.771593	-0.11736
Symptom_anemia	-0.10595	0.834	0.727625	0.808947	-0.08132
Symptom_menstrual pain	-0.10562	0.842	0.72828	0.809408	-0.08113
Symptom_PMS	-0.07073	0.26	0.70277	0.754281	-0.05151
Primary disease_PMS	-0.06672	0.164	0.700359	0.748683	-0.04832
Constant term	-0.05267	1	-	-	-

EQ-5D-5L, 5-level EQ-5D version; PMS, premenstrual syndrome

**Table:** c) Tobit Model (TB)

EQ-5D-5L utility score	Coefficient	SE	t	P > t	95% Confidence Interval	
Symptom_nausea/vomiting	-0.14612	0.019482	-7.5	0	-0.18443	-0.10782
Symptom_anemia	-0.13813	0.024307	-5.68	0	-0.18592	-0.09034
Symptom_menstrual pain	-0.10518	0.021099	-4.99	0	-0.14667	-0.0637
Symptom_PMS	-0.0899	0.020462	-4.39	0	-0.13014	-0.04967
Constant term	1.016435	0.022684	44.81	0	0.971833	1.061037

When the explanatory variable increases by 1, the latent variable of the EQ-5D-5L utility score can be interpreted as increasing by that coefficient

EQ-5D-5L, 5-level EQ-5D version; PMS, premenstrual syndrome; SE, standard error

**Table:** The difference in the EQ-5D-5L utility score is shown below as the mean of patient’s reduced EQ-5D-5L utility score

Variable description	Coefficient	Variable mean	X = 1	X = 0	Difference
Symptom_nausea/vomiting	-0.14612	0.249	0.64317	0.789292	-0.14612
Symptom_anemia	-0.13813	0.834	0.729978	0.86811	-0.13813
Symptom_menstrual pain	-0.10518	0.842	0.736289	0.841474	-0.10518
Symptom_PMS	-0.0899	0.26	0.68638	0.776283	-0.0899
Constant term	1.016435	1	-	-	-

EQ-5D-5L, 5-level EQ-5D version; PMS, premenstrual syndrome

### Development of predicting models

#### Step 1. Confirmation of the relationships between the objective variable and each explanatory variable

Relationships between the objective variable and each of the explanatory variables considered in the analysis were determined using a univariate regression analysis. The following 15 variables were found to be related to the EQ-5D-5L utility scores: “age” (p = 0.013), “age group (40 and over)” (p = 0.001), “drug_low dose oral contraceptives” (p = 0.019), “drug_iron preparation (IV)” (p = 0.037), “drug_estrogen preparation” (p = 0.012), “during menstrual period” (p < 0.001), “symptom_PMS” (p = 0.002), “symptom_menstrual pain” (p < 0.001), “symptom_menstrual symptoms” (p < 0.001), “symptom_anemia” (p < 0.001), “symptom_nausea” (p < 0.001), “symptom_vomiting” (p < 0.001), “symptom_nausea/vomiting” (p < 0.001), “nausea_severity” (p < 0.001), and “vomiting_frequency” (p < 0.001).

#### Step 2. Evaluation of multicollinearity

In the evaluation of correlation between the explanatory variables, for two variables with an absolute value of r greater than or equal to 0.7, one of them was selected from the viewpoint of clinical and versatility (Additional file 2). The variables with VIF greater than 5 were “symptom_nausea/vomiting”, “symptom_nausea”, “primary disease_dysmenorrhea/PMS”, “primary disease_all”, and “primary disease_dysmenorrhea”. However, for the three variables other than “symptom_nausea/vomiting” and “symptom_nausea”, no new variable selection was made because it was already conducted in the variable selection by correlation coefficients considering possibility of multicollinearity. The nested questions including “symptom_nausea/vomiting” and “symptom_nausea” were excluded from the evaluation in Step 2 because r between other variables cannot be calculated. These were re-evaluated in Step 3 to determine whether they could be explanatory variable candidates.

#### Step 3. Assessment of clinical validity

The three variables, “drug_low dose oral contraceptives”, “drug_estrogen preparation”, and “drug_others”, were excluded from the explanatory variable candidates because they were intervention factors and might be dependent on the variable related to the primary disease. “Symptom_menstrual pain” is a nested question for patients who responded that they were during the menstrual period. However, it was added to the explanatory variable candidates because menstrual pain was determined to obviously affect the QOL of the patients from a clinical perspective. For “symptom_nausea” and “symptom_nausea/vomiting,” for which multicollinearity was observed in Step 2, “symptom_nausea/vomiting” was selected as an explanatory variable candidate because 94 cases were found to overlap between patients with “symptom_nausea” (n = 94) and those with “symptom_nausea/vomiting” (n = 96).

The variables that were candidates for explanatory variables as a result of evaluation in Step 1 through Step 3 are shown in Additional file 3.

Table 2a), b), and c) summarize the results of the stepwise analysis using the following three statistical models (linear model, GLM, and Tobit model) with the EQ-5D-5L utility scores as objective variables.

### Goodness of fit

The results of the goodness-of-fit using the estimation dataset and validation dataset are summarized in Table 3). The model with the smallest MSE was GLM. The equation obtained by this analysis (all data) is as follows:

**Table 3:** EQ-5D-5L Goodness-of-fit results

Model	MAE	MSE	Spearman’s rho
LM	0.103793	0.019295	0.573
GLM	0.103786	**0.01926**	0.567
TM	0.107874	0.020417	0.551

Light yellow highlight indicates the model judged to be the best model based on each evaluation indicator, and bold indicates the model selected as the model with the smallest MSE.

EQ-5D-5L, 5-level EQ-5D version; GLM, Generalized Linear Model; LM, Linear Model; MAE, mean absolute error; MSE, mean squared error; TM, Tobit Model

𝑄𝑂𝐿=exp(− 0.05267 − 0.16500 * “symptom_nausea/vomiting” − 0.10595 * “symptom_anemia” − 0.10562 * “symptom_menstural pain” − 0.07073 * “symptom_PMS”− 0.06672 * “primary disease_PMS”).

Estimation with the GLM model indicated that the decrement of the EQ-5D-5L utility score (disutility) related to nausea/vomiting was − 0.117 in the patients taking iron preparations for HMB or anemia (Table 2b)).

## Discussion

In this study, we conducted a cross-sectional web-based survey involving 385 patients taking iron preparations for HMB or anemia (of these, nausea/vomiting were noted in 96). We established an estimating equation for predicting the EQ-5D-5L utility score by evaluating the QOL using the EQ-5D-5L questionnaire and collecting information on the presence of nausea/vomiting or other outcome measures. In addition, we assessed whether the presence of nausea/vomiting is a significant predictive factor for the EQ-5D-5L utility score in patients taking iron preparations for HMB or anemia. When establishing an estimating equation for predicting the EQ-5D-5L utility score, we examined the accuracy of the three statistical models. It was shown that estimation with GLM was the most appropriate. However, there were no marked differences in the degree of goodness-of-fit among the three statistical models. The explanatory variables selected as statistically significant variables were also almost always consistent.

The EQ-5D-5L utility score (mean ± SD) of 385 participants in this study was 0.79 ± 0.16. The score was lower than the reported EQ-5D-5L utility score (mean ± SD) in the age group of 40 to 49 years Japanese women’s values of 0.945 ± 0.090 [15]. As previously reported [4], HMB or anemia negatively impacted QOL utility score in this study as well.

Based on the estimating equation for predicting the EQ-5D-5L utility score, significant variables for the EQ-5D-5L utility score in patients taking iron preparations for HMB or anemia included “symptom_nausea/vomiting”, “symptom_anemia”, “symptom_menstrual pain”, “symptom_PMS”, and “primary disease_PMS”. These were evaluated as variables that significantly decrease the EQ-5D-5L utility score. In particular, “symptom_nausea/vomiting” was the most important factor that reduces the EQ-5D-5L utility score (coefficient: -0.165). Estimation with the GLM model indicated that the decrement of the EQ-5D-5L utility score (disutility) related to nausea/vomiting was − 0.117 in the patients taking iron preparations for HMB or anemia. To our knowledge, this is the first report to statistically evaluate that the presence of nausea/vomiting in patients taking iron preparations for HMB or anemia significantly reduces the EQ-5D-5L utility score, that is, QOL. Fujii et al. conducted a single-center retrospective descriptive study and evaluated the EQ-5D-5L utility values for patients receiving outpatient cancer chemotherapy in Japan [16]. They reported that EQ-5D-5L utility scores were significantly improved after pharmaceutical intervention for nausea and vomiting and decreasing the incidence of them from 0.8145 to 0.8603 (+ 0.0458 improved). As their study assessed changes in the EQ-5D-5L utility score before and after pharmaceutical intervention for nausea and vomiting in subjects differing from those of our study, it is not adequate to compare their results with those of this study. However, the negative effects of nausea/vomiting on the QOL may be marked.

The presence of menstrual pain was also shown to be an important factor that reduces the EQ-5D-5L utility score (coefficient: -0.10562). A study involving Japanese females evaluated the relationship between the EQ-5D-5L utility score and menstrual symptoms [17], and indicated that lower abdominal pain was negatively correlated (coefficient: -0.103). Therefore, the results of this study may be consistent from clinical aspects.

When establishing an estimating equation for predicting the EQ-5D-5L utility score, PMS remained as a significant explanatory variable that reduces the EQ-5D-5L utility score among the primary diseases (endometriosis, uterine myoma, adenomyosis uteri, endometrial polyp, dysmenorrhea, PMS, HMB, others) for the explanatory variables input to the stepwise method shown in Table 2b (coefficient: -0.07073). The EQ-5D-5L utility score in Japanese patients with PMS is reportedly 0.795 ± 0.120 (range: 0.362–0.949) [18]. Considering that only PMS remained as a significant explanatory variable among the primary diseases of the subjects in this study, it was shown that PMS more markedly influenced a reduction in the EQ-5D-5L utility score-based QOL compared with dysmenorrhea or HMB in females taking iron preparations.

It was indicated that the EQ-5D-5L utility score further decreased when symptoms of PMS were present (coefficient: -0.06672). However, the influence of differences in the severity of PMS on the EQ-5D-5L utility score could not be evaluated because this study was a questionnaire survey involving patients, and no data on severity have been collected. The relationship between the severity of PMS symptoms and EQ-5D-5L utility score should be further evaluated.

Several limitations in this study need to be considered. First, generalization of the patients in this study. As this was a web-based survey involving patients registered on the disease patient panel, the patient group’s representativeness may not have been sufficiently secured. However, the peak age of onset for IDA in females is 40 to 49 years [19]; therefore, considering the mean age (41.6 years) of the survey subjects, their representativeness may be secured to some degree. Secondly, this was a web-based survey involving patients, and there may have been a discrepancy between some answers, such as information on primary diseases, and physicians’ assessment or laboratory data. However, we made efforts to avoid wrong answers as much as possible by devising the expressions of questions and adding explanations. In addition, in the nature of a web-based survey involving patients, to avoid inaccurate information was collected, we did not ask the dosage/day, the frequency and the bland name of iron preparations. Thirdly, this study was a cross-sectional design. Patients taking iron preparations for HMB or anemia are not always taking them every day. Although the symptoms of nausea/vomiting may come and go, the presence or absence of nausea/vomiting at a point in time differentiates the patient’s condition, and the EQ-5D-5L utility score for that day is evaluated. In order to capture the QOL values of such a disease with fluctuating patient conditions and symptoms, the EQ-5D-5L survey with continuous assessment using a prospective cohort study design is more preferable. Lastly, in this study, external validation was not conducted due to the limited number of samples. However, we managed this by dividing the data obtained into estimation and validation datasets and performing cross validation.

## Conclusion

In this study, we collected information on the presence of nausea/vomiting, other patient reported outcomes and characteristics from 385 patients taking iron preparations for HMB or anemia, and established an estimating equation for predicting the EQ-5D-5L utility score. It was shown that the presence of nausea/vomiting significantly reduced the EQ-5D-5L utility score in patients taking iron preparations for HMB or anemia. The decrement of the value was estimated to be -0.117. Reproductive-aged women worldwide are frequently suffered by HMB or anemia. Not only does this in itself affect the QOL, but nausea and vomiting caused by taking iron preparations were also found to negatively impact QOL. Our society needs to recognize and address this problem.

### Electronic supplementary material

Below is the link to the electronic supplementary material.

Supplementary Material 1

Supplementary Material 2

Supplementary Material 3

## Data Availability

The datasets generated and/or analyzed during the study are available from the corresponding author on reasonable request.
