# Correction: Impact of nausea/vomiting on EQ-5D-5L utility scores in patients taking iron preparations for heavy menstrual bleeding or anemia

**DOI:** 10.1186/s12905-023-02689-2

**Published:** 2023-10-25

**Authors:** Kyoko Ito, Yuko Mitobe, Ryo Inoue, Mikio Momoeda

**Affiliations:** 1 Medical Affairs Department, Torii Pharmaceutical Co., Ltd., 3-4-1 Nihonbashi-Honcho, Chuo-ku, Tokyo, 103-8439 Japan; 2 Aiiku Maternal and Child Health Center, Aiiku Hospital, 1-16-10 Shibaura, Minato-ku, Tokyo, 105-8321 Japan

**Correction to: BMC Women’s Health (2023) 23:505**

10.1186/s12905-023-02652-1

In this article [1], the listed of the table 2 were in wrong order, now it has been updated.

The original article has been corrected.
