# Supplementary material for: Impact of nausea/vomiting on EQ-5D-5L utility scores in patients taking iron preparations for heavy menstrual bleeding or anemia
Source: BMC Womens Health. 2023 Sep 21;23:505. doi: 10.1186/s12905-023-02652-1 (PMC10512526; doi:10.1186/s12905-023-02652-1)
Supplement: Supplementary file 1 — Supplementary Material 1 [file 12905_2023_2652_MOESM1_ESM.docx]

**Additional file 1**

Descriptive statistics for questionnaire responses

**Journal name**

BMC Women’s Health

**Author information**

Kyoko Ito^1^, Yuko Mitobe^2^, Ryo Inoue^3^, Mikio Momoeda^4^

^1^ Medical Affairs Dept., Torii Pharmaceutical Co., Ltd., 3-4-1, Nihonbashi-Honcho, Chuo-ku, Tokyo 103-8439, Japan

^2^ Aiiku Maternal and Child Health Center, Aiiku Hospital, 1-16-10 Shibaura, Minato-ku, Tokyo 105-8321, Japan

**Corresponding author**

Mikio Momoeda, M.D., Ph.D.

Aiiku Maternal and Child Health Center, Aiiku Hospital

1-16-10 Shibaura, Minato-ku, Tokyo 105-8321, Japan

Tel: +81-3-6453-7300

Fax: +81-3-6453-73

E-mail: momoedam@gmail.com

**Additional file 1** Descriptive statistics for questionnaire responses

| Variable description | All | Presence of nausea/vomiting | | | | |  |  |
| --- | --- | --- | --- | --- | --- | --- | --- | --- |
|  | Total  N=385 | No  N=289 | | | Yes  N=96 | p-value |  |  |
| Sex (Female=1) | 385 (100.0%) | 289 (100.0%) | | | 96  (100.0%) | - |  |  |
| Age | 41.6  (7.7) | 42.3  (7.5) | | | 39.6  (8.0) | 0.002 |  |  |
| Age group  (40 and over=1) | 260  (67.5%) | 211  (73.0%) | | | 49  (51.0%) | <0.001 |  |  |
| Primary disease_all | 174  (45.2%) | 135  (46.7%) | | | 39  (40.6%) | 0.300 |  |  |
| Primary disease_endometriosis | 56  (14.5%) | 37  (12.8%) | | | 19  (19.8%) | 0.092 |  |  |
| Primary disease_uterine myoma | 130  (33.8%) | 103  (35.6%) | | | 27  (28.1%) | 0.180 |  |  |
| Primary disease_adenomyosis uteri | 35  (9.1%) | 26  (9.0%) | | | 9  (9.4%) | 0.910 |  |  |
| Primary disease_endometrial polyp | 6  (1.6%) | 1  (0.3%) | | | 5  (5.2%) | <0.001 |  |  |
| Primary disease_dysmenorrhea | 88  (22.9%) | 52  (18.0%) | | | 36  (37.5%) | <0.001 |  |  |
| Primary disease_PMS | 63  (16.4%) | 32  (11.1%) | | | 31  (32.3%) | <0.001 |  |  |
| Primary disease_HMB | 90  (23.4%) | 66  (22.8%) | | | 24  (25.0%) | 0.660 |  |  |
| Primary disease_others | 27  (7.0%) | 25  (8.7%) | | | 2  (2.1%) | 0.029 |  |  |
| Symptom_ dysmenorrhea/PMS | 116  (30.1%) | 70  (24.2%) | | | 46  (47.9%) | <0.001 |  |  |
| Drug_low dose oral contraceptives^a^ | 64  (16.6%) | | 42  (14.5%) | | 22  (22.9%) | 0.056 | |  |
| Drug_iron preparation (IV)^a^ | 39  (10.1%) | | 24  (8.3%) | | 15  (15.6%) | 0.039 | |  |
| Drug_iron preparation (oral)^a^ | 266  (69.1%) | | 207  (71.6%) | | 59  (61.5%) | 0.062 | |  |
| Drug_iron preparation (oral supplement)^a^ | 146  (37.9%) | | 99  (34.3%) | | 47  (49.0%) | 0.010 | |  |
| Drug_estrogen preparation^a^ | 71  (18.4%) | | 47  (16.3%) | | 24  (25.0%) | 0.056 | |  |
| Drug_others^a^ | 52  (13.5%) | | 39  (13.5%) | | 13  (13.5%) | 0.990 | |  |
| During menstrual period | 114  (29.6%) | | | 75  (26.0%) | 39  (40.6%) | 0.006 | | |
| Symptom_menstrual pain^b^ | 96  (84.2%) | | | 58  (77%) | 38  (97%) | 0.005 | | |
| Symptom_PMS | 100  (26.0%) | | | 70  (24.2%) | 30  (31.3%) | 0.170 | | |
| Symptom_menstrual symptoms | 214  (55.6%) | | | 145  (50.2%) | 69  (71.9%) | <0.001 | | |
| Symptom_anemia | 321  (83.4%) | | | 231  (79.9%) | 90  (93.8%) | 0.002 | | |
| Symptom_nausea | 94  (24.4%) | | | 0  (0.0%) | 94  (97.9%) | <0.001 | | |
| Symptom_vomiting | 27  (7.0%) | | | 0  (0.0%) | 27  (28.1%) | <0.001 | | |
| Symptom_nausea/vomiting | 96  (24.9%) | | | 0  (0.0%) | 96  (100.0%) | <0.001 | | |

The numbers in the table represent N (%) for discrete variables and mean (SD) for continuous variables. P-values represent the p-value resulting from the Pearson's chi-square test for discrete variables and the t-test for continuous variables.

^a^ Medication use was defined as having medication if responding that a patient had taken drugs at least within the past 3 months.

^b^ Only patients who are during the menstruating period can respond.

HMB, heavy menstrual bleeding; IV, intravenous; PMS, premenstrual syndrome; SD, standard deviation.
