# Supplementary material for: Impact of nausea/vomiting on EQ-5D-5L utility scores in patients taking iron preparations for heavy menstrual bleeding or anemia
Source: BMC Womens Health. 2023 Sep 21;23:505. doi: 10.1186/s12905-023-02652-1 (PMC10512526; doi:10.1186/s12905-023-02652-1)
Supplement: Supplementary file 2 — Supplementary Material 2 [file 12905_2023_2652_MOESM2_ESM.docx]

**Additional file 2**

Evaluation of correlation coefficient (r)

**Journal name**

BMC Women’s Health

**Author information**

Kyoko Ito^1^, Yuko Mitobe^2^, Ryo Inoue^3^, Mikio Momoeda^4^

^1^ Medical Affairs Dept., Torii Pharmaceutical Co., Ltd., 3-4-1, Nihonbashi-Honcho, Chuo-ku, Tokyo 103-8439, Japan

^2^ Aiiku Maternal and Child Health Center, Aiiku Hospital, 1-16-10 Shibaura, Minato-ku, Tokyo 105-8321, Japan

**Corresponding author**

Mikio Momoeda, M.D., Ph.D.

Aiiku Maternal and Child Health Center, Aiiku Hospital

1-16-10 Shibaura, Minato-ku, Tokyo 105-8321, Japan

Tel: +81-3-6453-7300

Fax: +81-3-6453-73

E-mail: momoedam@gmail.com

**Additional file 2** Evaluation of correlation coefficient (r)

Variable selections were performed for variables of |r| >0.7.

| Variable description | Correlation coefficient | Variable selection |
| --- | --- | --- |
| Age | 0.8293 | - “Age” (continuous quantity) was selected because it is easier to interpret. |
| Age group (40 and over=1) |  |  |
| Primary disease_uterine myoma | 0.7863 | - Since the impact on the EQ-5D-5L utility score is expected to differ depending on the type of disease, “primary disease_uterine myoma” which specifies the disease was selected. |
| Primary disease_all |  |  |
| Primary disease_dysmenorrhea | 0.8289 | - “Primary disease_dysmenorrhea” which is more disease specific was selected. |
| Symptom_ dysmenorrhea/PMS |  |  |
| Symptom_nausea | 0.9861 | - Evaluated in Step 3 |
| Symptom_nausea/vomiting |  |  |
| Nausea_duration | 0.9994 | - Evaluated in Step 3 |
| Vomiting_duration |  |  |

EQ-5D-5L, 5-level EQ-5D version; PMS, premenstrual syndrome.
