# Supplementary material for: Impact of nausea/vomiting on EQ-5D-5L utility scores in patients taking iron preparations for heavy menstrual bleeding or anemia
Source: BMC Womens Health. 2023 Sep 21;23:505. doi: 10.1186/s12905-023-02652-1 (PMC10512526; doi:10.1186/s12905-023-02652-1)
Supplement: Supplementary file 3 — Supplementary Material 3 [file 12905_2023_2652_MOESM3_ESM.docx]

**Additional file 3**

Candidates for explanatory variables for the EQ-5D-5L utility scores

**Journal name**

BMC Women’s Health

**Author information**

Kyoko Ito^1^, Yuko Mitobe^2^, Ryo Inoue^3^, Mikio Momoeda^4^

^1^ Medical Affairs Dept., Torii Pharmaceutical Co., Ltd., 3-4-1, Nihonbashi-Honcho, Chuo-ku, Tokyo 103-8439, Japan

^2^ Aiiku Maternal and Child Health Center, Aiiku Hospital, 1-16-10 Shibaura, Minato-ku, Tokyo 105-8321, Japan

**Corresponding author**

Mikio Momoeda, M.D., Ph.D.

Aiiku Maternal and Child Health Center, Aiiku Hospital

1-16-10 Shibaura, Minato-ku, Tokyo 105-8321, Japan

Tel: +81-3-6453-7300

Fax: +81-3-6453-73

E-mail: momoedam@gmail.com

**Additional file 3** Candidates for explanatory variables for the EQ-5D-5L utility scores

| Candidates for explanatory variables |
| --- |
| Age |
| Primary disease_endometriosis |
| Primary disease_uterine myoma |
| Primary disease_adenomyosis uteri |
| Primary disease_endometrial polyp |
| Primary disease_dysmenorrhea |
| Primary disease_PMS |
| Primary disease_HMB |
| Primary disease_others |
| Drug_iron preparation (IV)^a^ |
| Drug_iron preparation (oral)^a^ |
| Drug_iron preparation (oral supplement)^a^ |
| Symptom_PMS |
| Symptom_menstrual pain |
| Symptom_anemia |
| Symptom_nausea/vomiting |

^a^ Medication use was defined as having medication if responding that a patient had taken drugs at least within the past 3 months.

EQ-5D-5L, 5-level EQ-5D version; HMB, heavy menstrual bleeding; IV, intravenous; PMS, premenstrual syndrome.
